# Management of Persistent Epistaxis Using Floseal Hemostatic Matrix vs. traditional nasal packing: a prospective randomized control trial

**DOI:** 10.1186/s40463-017-0248-5

**Published:** 2018-01-08

**Authors:** Scott Murray, Adrian Mendez, Alexander Hopkins, Hamdy El-Hakim, Caroline C. Jeffery, David W. J. Côté

**Affiliations:** 1 University of Alberta, Faculty of Medicine, Edmonton, AB Canada; 2 Department of Surgery, Division of Otolaryngology-Head and Neck Surgery, University of Alberta, Edmonton, AB Canada

**Keywords:** Floseal® (Baxter, USA), Epistaxis, Nasal packing, Persistent

## Abstract

**Background:**

Epistaxis is the most common emergent consultation to otolaryngology-head & neck surgery (OHNS) and with 60% of the population having experienced an episode and 1.6 in 10,000 requiring hospitalization in their lifetime. In preliminary studies Floseal® (Baxter, USA) Hemostatic Matrix has shown efficacy in up to 80% of persistent anterior epistaxis. We sought to evaluate the clinical efficacy and cost-effectiveness of Floseal® (Baxter, USA) compared to traditional nasal packing for persistent epistaxis.

**Methods:**

A prospective, randomized controlled trial was conducted on all adult patients consulted to the OHNS service at the tertiary referral centers of the University of Alberta Hospital and Royal Alexandra Hospital for persistent epistaxis. Patients were randomized to the Floseal® (Baxter, USA) or traditional packing study arms. Our main clinical outcome measures were: 1) Hemostasis directly following treatment and at 48 h post-treatment, and 2) self-reported patient comfort at 48 h post-treatment. Further, trial data was used for a formal cost-effectiveness analysis to determine incremental cost-effectiveness ratio (ICER). Univariate sensitivity analysis and uncertainty analysis were performed.

**Results:**

There were no significant differences between groups for initial hemostasis (76.9% vs. 84.6%, *p* = 1.000) or, hemostasis at 48 h (76.9% vs. 69.2%, *p* = 1.000), requirement for admission (15.4% vs. 46.1%, *p* = 0.2016) or 30-day re-presentation rates (15.4% vs. 46.1%, *p* = 0.2016). Floseal® (Baxter, USA) was superior for decreased pain during placement (2.42 vs. 7.77, *p* = 0.0022), treatment (0.50 vs. 4.46, *p* = 0.0007) and removal (0 vs. 3.85, *p* = 0.0021). Floseal® (Baxter, USA) provides an average $1567.61 per patient savings from the single-payer system point of view and has an ICER of - $11,891 per re-bleed prevented (95% CI: -$37,658 to +$473). Uncertainty analysis shows that Floseal® has >90% chance of not only being cost-effective, but the dominant (preferred) treatment.

**Conclusions:**

Floseal® (Baxter, USA) was demonstrated to be an effective, comfortable and cost-effective alternative treatment of persistent epistaxis when compared to traditional packing methods for patients referred to OHNS with a normal coagulation profile.

**Trial registration:**

Trial registration number: NCT02488135. Date registered: June 26, 2015.

## Background

Epistaxis is a common problem with 60% of the population having experienced epistaxis in their lifetime and 6% of these individuals seeking medical care within a hospital [1]. Furthermore, 1.6 in 10,000 will require hospitalization [2]. There are a wide variety of nasal packing techniques available with the most common applied including Merocel ® (Medtronic, USA) packing, petroleum-infused gauze and inflatable balloons, such as Rapid rhino® (Smith & Nephew, UK). These techniques, although effective, may be accompanied by complications such as significant patient discomfort, infection, septal perforation, alar necrosis, and cardiovascular instability [3]. Therefore, alternatives to the traditional nasal packing method have been developed including dissolvable packing options such as hemostatic gelatin-thrombin matrices (eg. Floseal® (Baxter, USA)), Gelfoam® (Pfizer, USA) and Surgicel®(Ethicon, USA).

Floseal® (Baxter, USA) is a biodegradeable matrix hemostatic sealant with two primary components. The first component includes bovine-derived gelatin particles that swell providing a tamponade effect and a framework for platelet aggregation. The second component is human-derived thrombin that helps to accelerate clot formation. Floseal’s® hemophilic properties allow it to conform to both wet and irregular surfaces producing effective and rapid hemostasis on mucosal surfaces.

Floseal® (Baxter, USA) has demonstrated efficacy across a wide variety of surgical specialties including otolaryngology-head & neck surgery (OHNS) [4–20]. Notably, Floseal® (Baxter, USA) has demonstrated efficacy in the management of both anterior and posterior epistaxis [4, 13, 14]. Cote et al. in 2010 demonstrated achievement of hemostasis in 80% of patients with refractory epistaxis treated with Floseal® (Baxter, USA) who would have otherwise gone on to have surgical ligation. Further, these patients demonstrated comparable times to discharge as surgical patients and there were no adverse events recorded with many patients reporting superior comfort compared to traditional nasal packing.

Despite these promising results, the optimal first-line management of epistaxis remains debatable [21]. A randomized control trial directly comparing the efficacy of Floseal® (Baxter, USA) to traditional management for the control of persistent epistaxis in an OHNS patient population has yet to be performed. Our objective was to assess whether Floseal® (Baxter, USA) Hemostatic Matrix controls persistent epistaxis as effectively, with less discomfort and as cost effectively as traditional packing methods in adult patients consulted to the OHNS service.

## Methods

### Trial design

This was an open label prospective randomized controlled trial with a parallel study design and a 1:1 allocation ratio. There were no changes to methods following trial commencement.

### Participants

Patient recruitment continued from July 1st 2015 until March 1, 2017. Adult patients (>18 years of age), consulted to the OHNS service for persistent epistaxis from the University of Alberta Hospital (UAH) or the Royal Alexandra Hospital (RAH) tertiary care centers were included in this study. Persistent epistaxis was defined as having previously failed management from a non-OHNS physician. Patients having failed management from an otolaryngologist in the previous 48 h, who had previously diagnosed coagulopathies or were on anticoagulation other than prophylactic ASA 81 mg were excluded. The consulting physician identified patients for inclusion during the initial consultation and consent was obtained upon initial patient contact either on the ward or in the emergency department (ED). All patients were assessed for possible cauterization prior to packing as per the standard of care at the University of Alberta. Each patient was also provided an information booklet describing the study in detail prior to discharge that included researcher contact information if they chose to withdraw themselves from the study at any time.

### Intervention

Eligible patients had their previously failed nasal packing removed. Both the experimental and control groups had their nares suctioned of residual clots followed by application of two disposable cotton pledgets soaked in 1:1 mixture of 0.1% oxymetazoline and 4% topical lidocaine. The experimental group then had 5cm^3^ of Floseal® (Baxter, USA) applied transnasally or directly to the source of bleeding if identifiable. Each 5cm^3^ preparation of Floseal® (Baxter, USA) was constituted in concordance with the product monograph. The control group received conventional nasal packing as their treatment method, which included either Merocel® (Medtronic, USA) nasal tampons or Vaseline impregnated gauze. If initial packing failed further management was at the behest of the provider. The treatment providers for this study included OHNS residents from the University of Alberta who had all been trained in the application of Floseal® (Baxter, USA) prior to study recruitment. Patients in the traditional nasal packing group received prophylactic antibiotics during treatment duration. All patients were provided with prescriptions for post-intervention nasal hygiene regimens that included Rhinaris® (Pharmascience, CAN) nasal spray and nasal saline irrigation. Neither management with Floseal® (Baxter, USA) or traditional nasal packing were considered independent indications for admission.

### Outcomes

Our primary outcome was hemostasis that was recorded directly post-treatment and at 48 h post-treatment. Hemostasis was defined as termination of bleeding without the requirement for further intervention within the first 4 h following treatment. Our secondary outcomes included patient comfort at 48 h post-treatment. Patient pain scores were ascertained by means of a patient questionnaire that was applied to each patient at 48 h follow-up. This questionnaire involved a pre-validated 10-point visual analog scale (VAS) pertaining to packing placement, treatment and removal [22]. These questionnaires were administered either in person or by telephone. The same researcher applied all questionnaires using a pre-prepared script to avoid undue bias or coercion. Initial hemostasis, patient demographics, prescriptions and treatment type were recorded at the time of consultation. Patient comfort scores were recorded at 48 h following application of the patient comfort VAS questionnaire. Re-bleeding, admission rates and re-presentation rates were recorded at 48 h and 30 days post-treatment.

### Sample size

An a priori sample size was calculated using a non-inferiority limit (d) set at 25%, significance level (α) of 5% and power of 80%. We assumed that success in each group would be 93% based on the literature examining anterior nasal packing in ideal conditions and considering our selection criteria in the Floseal® (Baxter, USA) population [23, 24]. Attrition was assumed to be 0% due to the short duration of treatment. This yielded 26 participants with 13 patients in each study arm.

### Randomization, allocation concealment and implementation

Patients were randomized to either Floseal® (Baxter, USA) (experimental) or traditional nasal packing (control) groups with allocation concealed by using sealed envelopes stored in two separate treatment rooms at the UAH and RAH prior to patients being enrolled in the study. These envelopes were pre-prepared with the aid of a computerized random number sequence generator by a researcher who was removed from patient treatment and recruitment. Therefore, the direct care team had no control over the chosen treatment for the patients. Following the acquisition of consent, at the time of consultation, the treating physician unmasked these numbered envelopes sequentially, thus assigning each patient with a unique study identifier linked to their treatment group. This was an open-label trial as neither the direct care team or patient could be blinded to the applied treatment.

### Statistical analysis of clinical end-points

This was a non-inferiority trial. A non-inferiority design was chosen due to the anticipated improvement in patient comfort and potential decreased cost thus making the experimental arm a preferred option if non-inferiority can be demonstrated in terms of hemostasis. Further, based on existing literature we did not anticipate a significant difference in terms of efficacy between these two groups. All dichotomous categorical variables were analyzed by means of the Fisher’s exact test. Mean age, CCI and VAS pain scores were compared using the non-parametric Mann-Whitney U Test.

### Economic evaluation

A trial-based economic evaluation was performed using clinical outcomes data with concurrent tracking of resource utilization throughout the trial. A micro-costing method was used to capture costs. This means that for each patient, costs were tabulated based on the actual number of units of each resource used (see Table 1). The societal perspective was used, which means that health care costs and patient productivity losses due to time off from work were collected concurrently. However, the data is presented in disaggregated form to show the perspectives of a single-payer health system (i.e. Alberta Health Services) and patients and their caregivers (Table 2). Costs included physician fees, surgical fees, in-patient surgical ward and medical ward costs, outpatient drug costs, and cost of nasal packing material. Since the study was mainly increased in incremental differences in costs, the initial cost of emergency room visits, ambulance costs, and emergency room physician fees were not included as they are assumed to be the same for both groups. In addition, only admission directly related to the episode of epistaxis were included to ensure that only disease-specific healthcare costs were included. Sources of cost data included: Alberta Health Services (AHS) physician fee schedule, drug benefits, operating room supply purchase prices, and the Canadian Institute for Health Information. All costs were expressed in Canadian 2016 dollars. The time-horizon was 30 days and the main clinical-outcome of interest was re-bleed rate at 48 h. Due to the short time-horizon, costs were not discounted.

**Table 1:** Key Unit Costs to Value Resource Use During Trial (2016 Canadian Dollars)

	Resource	Unit	Unit Cost (2016 Canadian Dollars)	Source
Acute care	Physician Consult	Fixed	78.21	AHS Medical Benefits Procedure List(Version April 1st, 2016)
	Physician procedure (control of epistaxis)	Fixed	125.00	
	Surgical ward	Day	994.0	AHS Financial Fiscal 2016
	Medical ward	Day	1164	
	Merocel®	Each	28.50	OR Bulk price
	Vaseline gauze	Each	8.50	
	Rapid rhino®	Each	180.00	
	Floseal®	Per 5 mL	326.83	
Convalescence and Follow-up	Salinex	Per mL	0.15	Alberta BlueCross Drug Price List (Version 2016)
	Polysporin	Per 28.3 g tube	2.94	
	Oral cephalexin 250 mg tablet	Per tablet	0.40	
	Oral clindamycin 300 mg tablet	Per tablet	0.44	
	Physician follow-up	Fixed	39.14	AHS Medical Benefits Procedure List (Version April 1st, 2016)
Productivity Costs	Wages lost	Per day	405.79	Government of Canada 2016 Census data

**Table 2:** Summary of self-reported comfort scores rated on a 10-point VAS at 48 h post-treatment comparing treatment (Floseal® (Baxter, USA)) to control (traditional packing methods)

Pain	Treatment (Floseal) (*N* = 13)	Control (*N* = 13)	Z-score	*p*-value^a^
Placement	2.4	7.8	3.6987	0.0022
Treatment	0.5	4.5	3.3800	0.0007
Removal	0	3.9	3.0699	0.0021

^a^ α = 0.05, significant results in bold using unpaired Mann-Whitney U test; mean 10-pt VAS of patient pain experienced (0 = no pain, 10 = worst pain ever experienced)

STATA 14.2 (StataCorp LP, TX) was used for all statistical analyses. Both univariate (i.e. price of Floseal® (Baxter, USA), Table 3) deterministic sensitivity analysis and non-parametric boot strapping of 1000 simulated patients was performed to generate 95% confidence interval for mean cost and effect differences between the Floseal® (Baxter, USA) group and control group for both the health system and societal perspectives (Fig. 3) [25]. This is a standard method of providing confidence intervals for data that is not normally distributed (i.e. costs).

**Table 3:** Uni-variate Sensitivity Analysis of Health-System Cost savings (by % Change in price of Floseal® (Baxter, USA))

Price Increase	0%	50%	100%	200%	300%	400%
Cost Savings	$1567.61	$1404.20	$1240.78	$913.95	$587.12	$260.29

The mean difference in costs was divided by the mean difference in effectiveness to generate an Incremental Cost-effectiveness Ratio (ICER) of cost in dollars per re-bleed avoided. To account for uncertainty due to sampling variation, a confidence ellipse graph was plotted along the cost-effectiveness plane. This graph demonstrates the quadrant where 50%, 75% and 95% of calculated ICERs will reside. Finally, a cost-effectiveness acceptability curve was plotted showing the probability that Floseal® (Baxter, USA) is cost-effective for a given threshold set by the decision-maker [26].

## Results

### Recruitment and participant flow diagram

**Fig. 1:**
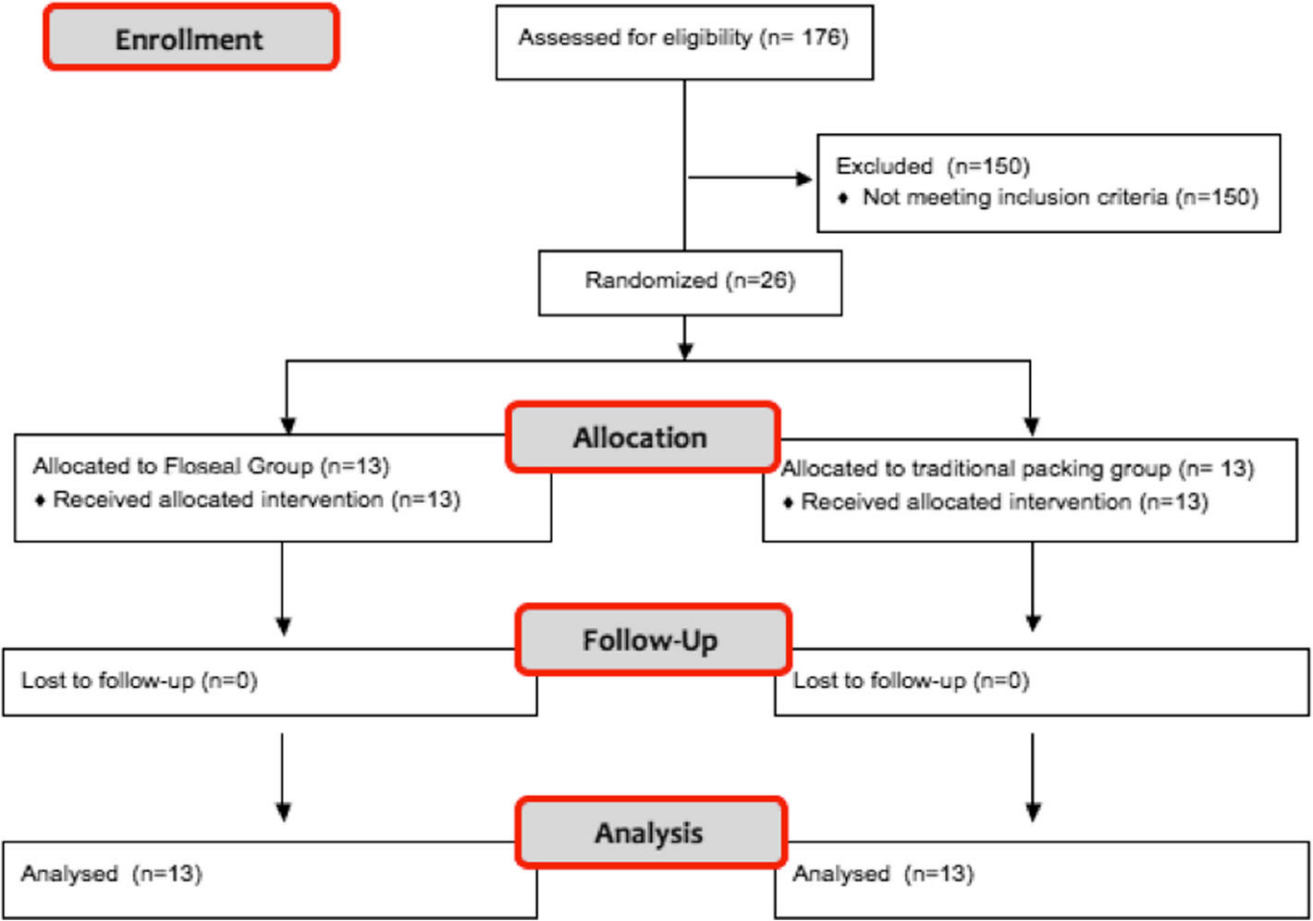
Patient flow diagram participant numbers for enrollment, allocation, follow-up and analysis

Patient recruitment continued from July 1st 2015 until March 1st, 2017 and is summarized in Fig. 1. Out of 176 eligible patients, a total of 26 patients participated in this study with 13 patients in each arm. All patients received the allocated intervention, no patients were lost to follow-up and all were analyzed. The trial was ended at a pre-determined 20-month end-point.

### Patient demographics

Patient demographics are summarized in Table 4. Mean age was 59.0 (range: 22.6-95.3) for Floseal® (Baxter, USA) and 55.1 (range:19.1-86.1) for traditional packing (*p* = 0.6101). Males comprised 61.5% for Floseal® (Baxter, USA) compared to 46.2% for the traditional packing group (*p* = 0.6951). The Floseal® (Baxter, USA) group had 15.38% of patients taking daily ASA 81 mg compared to 23.07% in the traditional packing group (*p* = 1.000). Lastly, the Charlson Comorbidity Index (CCI) was calculated to be the same between Floseal® (Baxter, USA) and traditional packing (2.3 vs. 2.3, *p* = 0.8966).

**Table 4:** Summary of baseline demographic and clinical characteristics comparing treatment (Floseal® (Baxter, USA)) to control (traditional packing methods)

	Treatment (Floseal) (*N* = 13)	Control (*N* = 13)	Z-score	*p*-value^a,b^
Age	59.0	55.1	0.5128	0. 6101^a^
Gender (Male)	8 (61.5%)	6 (46.2%)	NA	0.6951^b^
ASA	2 (15.4%)	3 (23.1%)	NA	1.000^b^
CCI	2.3	2.3	−0.1282	0.8966^a^

^a^ α = 0.05, significant results in bold using unpaired Mann-Whitney U test, *CCI* Charlson Comorbidity Index, *ASA* Aspirin 81 mg po daily

^b^ α = 0.05, significant results in bold using Fisher’s exact test

### Hemostasis, admission rates and adverse events

Our data on hemostasis, admission rates and adverse events are summarized in Table 5. When comparing between the Floseal® (Baxter, USA) and control groups, there were no statistically significant differences for hemostasis initially (10 (76.9%) vs. 11 (84.6%) respectively, *p* = 1.000), hemostasis at 48 h (10 (76.9%) vs. 9 (69.2%) respectively, *p* = 1.000), requirement for admission (2 (15.4%) vs. 6 (46.1%) respectively, *p* = 0.2016) or 30-day re-presentation rates (2 (15.4%) vs. 6 (46.1%), p = 0.2016). There were no adverse events recorded related to the nasal packing in either the Floseal® (Baxter, USA) or packing groups (0 (0.0%) vs. 0 (0.0%), *p* = 1.0000).

**Table 5:** Summary of hemostasis, admission and re-presentation rates comparing treatment (Floseal® (Baxter, USA)) to control (traditional packing methods)

	Treatment (Floseal) (*N* = 13)	Control (*N* = 13)	*p*-value^a^
Hemostasis Post-Treatment	10 (76.9%)	11 (84.6%)	1.0000
Hemostasis at 48-h	10 (76.9%)	9 (69.2%)	1.0000
Admission Required	2 (15.4%)	6 (46.1%)	0.2016
30 Day ED Re-Presentation	2 (15.4%)	6 (46.1%)	0.2016
Adverse Events	0 (0.0%)	0 (0.0%)	1.0000

^a^ α = 0.05, significant results in bold using Fisher’s exact test

### Patient comfort

Our data on mean self-reported comfort scores rated on a 10-point VAS are summarized in Table 2. Floseal® (Baxter, USA) was superior for pain during placement (2.4 vs. 7.8, *p* = 0.0022), treatment (0.5 vs. 4.5, *p* = 0.0007) and removal (0.0 vs. 3.9, *p* = 0.0021). There were only two patients within the cohort who received Vaseline gauze packing and, although the sample size is too small to statistically compare, their mean pain scores for placement (6.5), treatment (5.0) and removal (3.0) were similar to the mean pain scores recorded for the entire traditional packing group.

### Economic evaluation

**Fig. 2:**
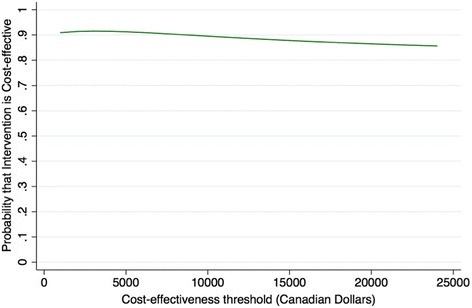
Cost effectiveness acceptability curve for Floseal® (Baxter, USA) Hemostatic Matrix

Table 1 provides a summary of the key sources of resource utilization in the trial separated by the two comparators in the study. Table 6 provides the mean costs, obtained from averaging individual patient costs for each group. From a single-payer, health system point of view, Floseal® (Baxter, USA) provides a mean cost-savings of $1567.61. When the larger societal perspective is taken into account, mean savings of $2233.36 are present due to the decreased work-productivity losses (Table 6). Univariate sensitivity analysis shows that even at 4-times the current price, treating with Floseal® (Baxter, USA) provides cost-savings (Table 3). An ICER of - $11,891 per re-bleed prevented (95% CI: -$37,658 to +$473) was obtained by bootstrap method. The negative ICER indicates a cost-savings. The cost-effectiveness acceptability curve (CEAC) also demonstrates that for a large range of treatment costs, Floseal® (Baxter, USA) maintains greater than 90% probability of being cost-effective. Furthermore, confidence ellipse plots for analysis from both the single-payer health system and societal perspectives (Fig. 2) show that Floseal® (Baxter, USA) is not only cost-effective, but the dominant intervention (i.e. it resides in the lower right quadrant).

**Table 6:** Comparison of Costs Between Floseal® (Baxter, USA)® and Usual Care

			Control (*N* = 13)		Treatment (Floseal) (*N* = 13)	
			Mean	Median (IQR)	Mean	Median (IQR)
Societal erspective	Health System Perspective	Direct disease-related health services (e.g. physician fees, material costs, admission, surgical fees, medications)	$2704.51	(354.71 – 3846.71)	$1136.90	(530.11 – 580.92)
	Non-Health Sector Costs	Work productivity losses (e.g. lost wages)	$1342.23	(405.79-1623.16)	$676.32	(405.79-405.79)
Total Costs			$4046.74	(1033.88-5469.87)	$1813.22	(945.10-1164.20)
Differential Mean Costs^a^ (95% CI)^b^			Health system perspective: -$1567.61 (−3787.25 to 652.00) Societal perspective: - $2233.36 (−5443.27 to 976.56)			

^a^ Floseal® (Baxter, USA) minus control

^b^ 95% non-parametric confidence interval based on 1000 bootstrap replications

## Discussion

Based on age, gender, ASA use and co-morbidities as represented by the CCI both the Floseal® (Baxter, USA) and traditional packing groups showed no significant differences. There were also no adverse events recorded related to either the Floseal® (Baxter, USA) or traditional packing treatments during the course of this study.

With a 77% rate of hemostasis the effectiveness of Floseal® (Baxter, USA) in our study was comparable to previous studies examining its application in the management of anterior and posterior epistaxis [4, 13, 14]. Cote et al. were able to achieve hemostasis in 80% of cases with comparable time to discharge as surgical management [4]. Similarly, Kilty et al. 2014 were able to achieve hemostasis in 80% of posterior epistaxis cases [13]. Mathiason and Cruz 2005 demonstrated that Floseal® (Baxter, USA) was rated as more effective, easier to use and more satisfying than traditional nasal packing. There were also fewer re-bleeds and otolaryngology consultations in the follow-up period [14].

There were no significant differences between groups for either hemostasis directly following treatment (10 (76.9%) vs. 11 (84.6%), *p* = 1.000), or at 48 h follow-up (10 (76.9%) vs. 9 (69.2%), *p* = 1.000). This would suggest that Floseal® (Baxter, USA) is an effective alternative as compared to traditional nasal packing in the management of persistent epistaxis. While a relative trend towards higher failures in initial hemostasis for Floseal® (Baxter, USA) may caution its use as the first line choice, the inverse trend was seen during the 48 h post-treatment with more re-bleeds occurring in the traditional packing group in that time interval. This difference may be attributed to the intranasal mucosal trauma caused by the insertion and removal of the traditional packs that is avoided with Floseal® (Baxter, USA).

Floseal® (Baxter, USA) was shown to be significantly less painful than traditional packing methods during placement (2.4 vs. 7.8, *p* = 0.0022), treatment (0.5 vs. 4.5, *p* = 0.0007) and removal (0.0 vs. 3.9, *p* = 0.0021). In light of the finding of non-inferiority, as described above, these pain scores provide the greatest support for Floseal® (Baxter, USA) as a first line therapy for the management of persistent epistaxis. The psychosocial toll of the often-prolonged discomfort involved with nasal packing is difficult to quantify but should not be overlooked. Similar to our results, both Kilty and Mathiason found that Floseal® (Baxter, USA) was reported as significantly more comfortable than traditional packing methods [13, 14]. However, this study is the first to include self-reported patient pain scales for the three phases of packing (placement, treatment and removal).

**Fig. 3:**
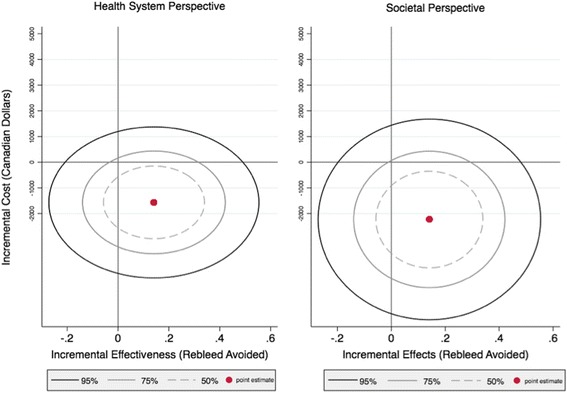
95% Confidence ellipses of bootstrapped incremental costs and incremental effects from both health system and societal perspectives (cost-effectiveness plane). Legend: *point estimate residing in the right lower quadrant indicates that the treatment (i.e. Floseal® (Baxter, USA)) is by definition, cost-effective and dominant)

Kilty et al. 2014 found that Floseal® (Baxter, USA) was more cost-effective than endoscopic surgery, posterior packing or embolization [13]. In this study, the economic evaluation was performed using “usual care” as the comparison group. This includes a variety of disposable products such as Merocel® (Medtronic, USA), petroleum infused gauze, and Rapid rhino® (Smith & Nephew, UK). Our analysis shows that Floseal® (Baxter, USA) provides significant cost-savings from a health system perspective and an even greater cost-savings when non-health sector work productivity losses were taken into account. Statistical bootstrapping and uncertainty analysis was used to provide probabilistic boundaries for interpreting our findings. Specifically, the cost-effective acceptability curve shows that Floseal® (Baxter, USA) remains cost-effective for a large range of total treatment costs. That is, decision-makers from different provinces with different willingness to pay for epistaxis management would find that Floseal® (Baxter, USA) has a > 90% chance of being cost-effective. These results are affirmed by the confidence ellipse plots along the cost-effectiveness plane (Fig. 3), which suggest that Floseal® (Baxter, USA) is not only cost-effective but it is the dominant intervention as it is both cheaper and more effective.

From a health system perspective the relatively greater, albeit non-significant, number of admissions (2 (15.4%) vs. 6 (46.1%), *p* = 0.2016) in the traditional packing group was the primary reason for this cost savings. Additionally, the primary increase in costs from a societal perspective arose from the requirement to re-present to the ED for the traditional packing removal. The decisions for admission were made independently of the management method, as packing on its own was not an indication for admission in either group. All reasons for admission are listed in Table 7. Other less significant costs in the traditional nasal packing group were prophylactic antibiotic requirements and increased analgesia prescriptions.

**Table 7:** Reasons for admission

Reason for Admission	Number of Patients	
	Treatment (Floseal) (*N* = 13)	Control(*N* = 13)
>2 previous packing attempts^a^	0	1
Pneumonia	0	1
Hemoglobin <70	0	1
New onset a-fib	1	0
Cardiac Surgery	0	1
Systolic Blood Pressure > 180	0	1
Sphenopalatine artery ligation	1	1

^a^Previous packing by non-OHNS clinician

Limitations of the study include the impossibility for blinding the physician investigator or the patient, and the inherent biases that this can present when using self-reported pain outcomes. We alsolimited our follow-up to 30 days thus limiting the analysis of outcomes or adverse events beyond this point. Further, 150 patients were excluded following consultation to our service, with only 26 being included. This is a product of our strict exclusion criteria specifically excluding those with bleeding diathesis or those on antiplatelet or anticoagulation therapy, which accounts for all patient exclusions. This was implemented to avoid the confounding nature of the widely variable coagulation profiles of patients consulted to our service for epistaxis thus improving population homogeneity. However, this strict control does reduce the study’s generalizability within the otolaryngology population. Lastly, a potential weakness of our study was its relatively high inferiority limit; the study team chose this limit due to the large anticipated improvement in patient pain scores thus allowing for a 25% difference in efficacy between study groups. Floseal® (Baxter, USA) was chosen as our intervention due to our center’s previous pilot study demonstrating the efficacy and safety of this particular product [4]. Future studies should focus on comparing Floseal® (Baxter, USA) to other dissolvable packing options.

## Conclusion

This is the first randomized controlled trial comparing Floseal® (Baxter, USA) Hemostatic Matrix to traditional nasal packing methods for patients requiring consultation to otolaryngology. Further, it is the first study to include a formal economic analysis when examining the role of Floseal® (Baxter, USA) for the management of epistaxis. The results of this study suggest that Floseal® (Baxter, USA) Hemostatic Matrix is an effective, comfortable and cost-effective alternative treatment of persistent epistaxis when compared to traditional packing methods for patients referred to OHNS with a normal coagulation profile.

## Abbreviations

AHS: Alberta Health Services
ASA: Aspirin
CCI: Charlson Comorbidity Index
CEAC: Cost-effectiveness acceptability curve
CI: 95% Confidence Interval
ED: Emergency department
Floseal® (Baxter, USA): Floseal® (Baxter, USA) Hemostatic Matrix
ICER: Incremental cost effectiveness ratio
OHNS: Otolaryngology-head & neck surgery
RAH: Royal Alexandra Hospital
UAH: University of Alberta Hospital
VAS: Visual Analog Scale
